# MiR-433-3p suppresses cell growth and enhances chemosensitivity by targeting CREB in human glioma

**DOI:** 10.18632/oncotarget.13789

**Published:** 2016-12-03

**Authors:** Shupeng Sun, Xiuyu Wang, Xinnv Xu, Hui Di, Jixiang Du, Bin Xu, Qiong Wang, Jinhuan Wang

**Affiliations:** ^1^ Tianjin Key Laboratory of Cerebral Vascular and Neurodegenerative Diseases, Tianjin Neurosurgical Institute, Department of Neurosurgery, Tianjin Huanhu Hospital, Tianjin 300350, China; ^2^ The Graduate School, Tianjin Medical University, Tianjin 300070, China; ^3^ Key Laboratory for Critical Care Medicine of the Ministry of Health, Tianjin First Center Hospital, Tianjin 300192, China; ^4^ Department of Neurosurgery, Affiliated Hospital of Hebei University, Baoding 071000, China

**Keywords:** miR-433-3p, CREB, glioma, carcinogenesis, chemosensitivity

## Abstract

Previous studies reported that miR-433 exerts function widely in human tumorigenesis and development. Here, we further investigate the potential role of miR-433 in glioma. Quantitative real-time PCR demonstrated that miR-433-3p and miR-433-5p were low expressed in glioma tissues and cell lines. Functional studies suggested that the overexpression of miR-433-3p suppressed proliferation, induced apoptosis and inhibited invasion and migration of human glioma cells. But the growth and metastasis of glioma cells were not significantly influenced by overexpression of miR-433-5p. In a xenograft model, we also showed that miR-433-3p had an inhibitory effect on the growth of glioma. Bioinformatics coupled with luciferase and western blot assays revealed that CREB is a direct target of miR-433-3p, and the overexpression of CREB can rescue the phenotype changes induced by miR-433-3p overexpression. Besides, miR-433-3p could increase chemosensitivity of glioma to temozolomide by targeting CREB *in vitro* and *in vivo*. Taken together, these results suggest that miR-433-3p may function as a potential marker in diagnostic and therapeutic target for glioma.

## INTRODUCTION

Glioma is the most common malignant tumor of central nervous system with rapid growth, low survival rate, and high mortality, especially to the patients who could not completely remove the cancer [1]. Despite great efforts made in the past several decades toward improvement of combined therapies for glioma, the prognosis remains grim [2]. A better understanding of the molecular mechanism of gliomagenesis and finding genes associated with the pathogenesis of glioma will lead to the development of novel targeted therapies for glioma. MicroRNAs (miRNAs) are a new class of endogenous, small (19~22nt) non-coding single-stranded RNA molecules that negatively regulate protein-coding genes by base pair matching with the 3′ untranslated region (UTR) of mRNA, resulting in that the translation of target gene is suppressed or the mRNA of target gene is degraded [3, 4]. MiRNAs can affect tumor development through a variety of mechanisms [5]. Abnormalities of miRNAs expression are now known to be involved in gliomagenesis and may function as oncogenes or tumor suppressors [6].

MiR-433 exerts diverse functions in human tumorigenesis and development [7–15]. It has been reported that miR-433 was down-regulated in gastric carcinoma [7], visceral adipose tissue of patients with non-alcoholic steatohepatitis [8] and hepatitis B virus-associated hepatocellular carcinoma (HCC) [9]. Moreover, microRNA-433 negatively regulates the expression of thymidylate synthase (TYMS) responsible for 5-fluorouracil sensitivity in HeLa cells [10]. Besides, microRNA-433 inhibits hepatocellular carcinoma cell migration in HCC [11] and suppresses hematopoietic cell growth and differentiation in myeloproliferative neoplasms [12]. A cohort of genes which contain GRB2 [7], HDAC6 [13] and GBP2 [12] have been identified and validated as targeted genes of miR-433. However, the role of miR-433 in human malignant glioma remains largely unknown. The aim of this study was to determine expression levels of miR-433 in glioma and to further investigate the potential role of miR-433 in this malignancy.

## RESULTS

### MiR-433-3p and miR-433-5p are down-regulated in malignant glioma

**Figure 1:**
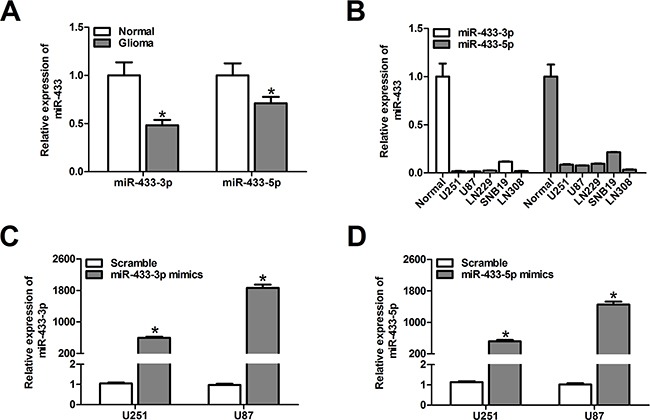
MiR-433-3p and miR-433-5p are down-regulated in malignant glioma **A**. The relative miR-433-3p and miR-433-5p expression levels in 6 normal brain tissues and 12 glioma tissues were analyzed by quantitative RT-PCR. *P < 0.05 versus Normal. **B**. Real-time PCR analysis of miR-433-3p and miR-433-5p expression in various glioma cell lines (U251, U87, LN229, SNB19 and LN308). **C. and D**. The efficiency of miR-433-3p and miR-433-5p mimics in U251 and U87 cells was confirmed by quantitative RT-PCR. *P < 0.05 versus Scramble. All experiments were repeated three times. All data are shown as mean ± SD.

To evaluate the expression pattern of miR-433-3p and miR-433-5p in malignant glioma, quantitative RT-PCR was used to detect the expression level in 12 malignant glioma tissues, 6 unmatched nonneoplastic brain specimens, and 5 glioma cell lines (U251, U87, LN229, SNB19 and LN308). We found that miR-433-3p and miR-433-5p expression in malignant glioma was significantly lower than in nontumor brain tissues (*P* < 0.01, Figure 1A). Similarly, 5 glioma cell lines showed markedly lower levels of miR-433-3p and miR-433-5p expression when compared with nontumor brain tissues (Figure 1B).

To elucidate the role of miR-433 in human glioma development, commercially synthesized miR-433-3p and miR-433-5p mimics were used to alter the levels of miR-433-3p and miR-433-5p in U251 and U87 glioma cells. The alteration of miR-433-3p and miR-433-5p was confirmed by quantitative RT-PCR. As shown in Figure 1C and 1D, the expression levels of miR-433-3p and miR-433-5p in U251 and U87 cells transfected with mimics were significantly elevated (*P* < 0.05).

### MiR-433-3p suppresses malignant behavior of glioma cells

**Figure 2:**
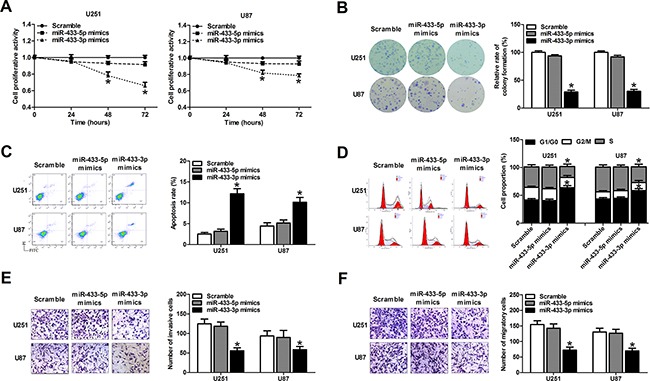
MiR-433-3p suppresses malignant behavior of glioma cells **A**. MTT assay of U251 and U87 cells transfected with miR-433-3p or miR-433-5p mimics was used to investigate the effect of miR-433 on cell growth. **B**. Cell clonogenicity was measured by the colony formation assay of U251 and U87 cells transfected with miR-433-3p or miR-433-5p mimics. **C**. Apoptosis in U251 and U87 cells was measured by Annexin V-FITC/ PI staining following transfection of miR-433-3p or miR-433-5p mimics. **D**. After transfection of miR-433-3p or miR-433-5p mimics to U251 and U87 cells, the cell cycle distribution was analyzed by flow cytometry. **E. and F**. The effect of miR-433-3p or miR-433-5p on invasion and migration in U251 and U87 cells was assessed by *in vitro* transwell assay. Representative images are shown on the left, and the average number of cells per field is shown on the right. All experiments were repeated three times. All data are shown as mean ± SD. *P < 0.05 versus Scramble.

The effects of miR-433 alteration on cell viability and growth were determined in glioma cells using MTT and colony formation assays. Overexpression of miR-433-3p remarkably inhibited cell viability at 48 h and 72 h after transfection compared with miR-433-5p mimic group and the scramble group in the U251 and U87 cell lines (*P* < 0.01, Figure 2A). The colony formation assay revealed that the colony formation rates of U251 and U87 cells transfected with miR-433-3p mimics were lower than in corresponding cells in the miR-433-5p mimic group and the scramble group (*P* < 0.05, Figure 2B).

To measure the effect of miR-433 on glioma cell apoptosis, we transfected miRNA mimics into U251 and U87 glioma cells, and assessed the ratio of apoptosis 48 h after transfection using flow cytometry. As a result, overexpression of miR-433-3p rather than miR-433-5p significantly induced apoptosis in both U251 and U87 cells (*P* < 0.01, Figure 2C). The cell cycle was also evaluated 48 h after mimics transfection by flow cytometry. As shown in Figure 2D, miR-433-3p rather than miR-433-5p delayed the progression of the cell cycle and inhibited cell proliferation by arresting the tumor cells at G0/G1 phase (*P* < 0.05). These results demonstrate that miR-433-3p inhibits the growth of glioma cells, while miR-433-5p had no significant effect on cell proliferation.

In addition, we analyzed the effects of miR-433 on cell invasion and migration in U251 and U87 cell lines by transwell assay. The results showed that cell invasion and migration were attenuated in miR-433-3p group compared with miR-433-5p group and scrambled group (*P* < 0.05, Figure 2E and 2F). These results indicate that miR-433-3p suppresses metastasis, thus functioning as a tumor suppressor in human glioma cells.

### CREB is a direct target of miR-433-3p

**Figure 3:**
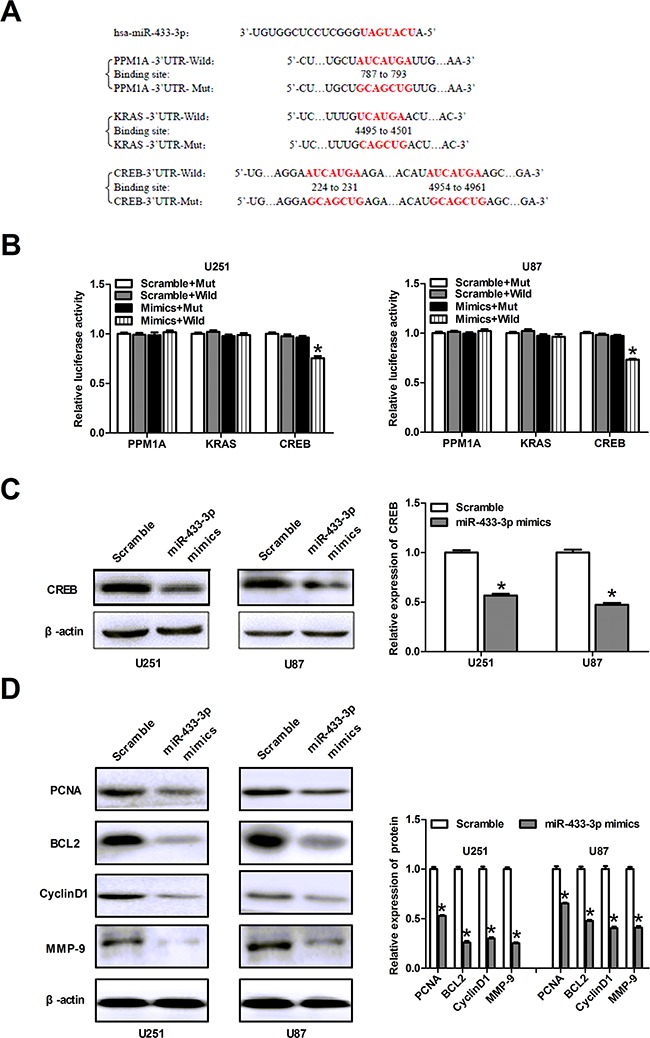
CREB is a direct target of miR-433-3p **A**. The sequence of miR-433-3p binding sites within PPM1A, KRAS and CREB. The reporter constructs of the PPM1A, KRAS and CREB 3′-UTR sequences and the mutated 3′-UTR sequences are shown in the schematic diagram. **B**. Luciferase reporter assay was performed to detect the relative luciferase activities of wild and mut PPM1A, KRAS or CREB reporters. *P < 0.05 versus Scramble + Mut. **C**. Western blot was used to detect the CREB protein level in U251 and U87 cells over-expressing miR-433-3p. *P < 0.05 versus Scramble. **D**. The relative expression levels of several downstream molecules of CREB, including PCNA, BCL2, MMP-9 and CyclinD1 were detected by immunoblotting analysis. *P < 0.05 versus Scramble. All experiments were repeated three times. All data are shown as mean ± SD.

To determine the mechanism underlying the inhibitory effects of miR-433-3p on glioma, the identification of the miR-433-3p downstream target genes is essential. Using TargetScan, PicTar, and miRanda, we predicted multiple putative targets of miR-433-3p based on the conserved seed region between miR-433-3p and the 3′-UTR of each gene (CREB, PPM1A and KRAS) (Figure 3A). We cloned the 3′-UTRs of three genes into the respective luciferase reporters. The results of luciferase reporter assay showed that relative activities of plasmid luciferase in U251 and U87 cells were not obviously changed in PPM1A groups and KRAS groups. But the group with the wild-type 3′-UTR of CREB showed markedly reduced luciferase activity in miR-433-3p mimics group compared with scramble miRNA group (*P* < 0.01, Figure 3B). Therefore, CREB may be a target gene of miR-433-3p. Furthermore, western blot was used to assess the effects of miR-433-3p on CREB expression. We transfected miR-433-3p mimics into U251 and U87 cells and found that overexpression of miR-433-3p reduced CREB protein expression (*P* < 0.05, Figure 3C). Taken together, these results suggest that CREB is a direct target gene of miR-433-3p.

In order to further decipher the mechanism related to the role of miR-433-3p, we examined the relative expression levels of several downstream proteins of CREB, including PCNA, BCL2, MMP-9 and CyclinD1 by western blot. After transfection with miR-433-3p mimics, the expression of PCNA, BCL2, MMP-9 and CyclinD1 in U251 and U87 cells were significantly decreased as compared to scrambled cells (*P* < 0.05, Figure 3D). These results indicate that miR-433-3p plays a critical role in abrogating the malignant phenotype of glioma.

### CREB can rescue the phenotypes caused by miR-433-3p

**Figure 4:**
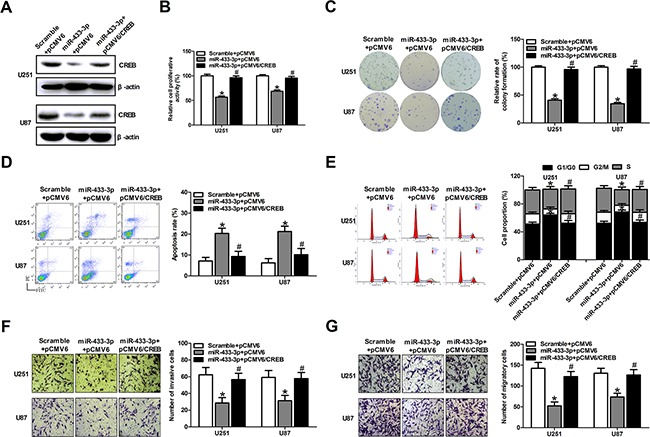
CREB can rescue the phenotypes caused by miR-433-3p **A**. Western blot analysis of CREB in U251 and U87 cells co-transfected with either miR-433-3p mimics or scramble and pCMV6/CREB or pCMV6 empty vector. **B. and C**. The transfected cells were submitted to MTT and colony formation assays to test the proliferation of U251 and U87 cells after co-transfection with mimics and vectors. **D. and E**. Apoptosis and cell cycle analysis were employed in U251 and U87 cells co-transfected with miR-433-3p mimics and pCMV6/CREB or their control oligomers and vectors. **F. and G**. Cell motility was examined by transwell invasion and migration assay after co-transfection with mimics and vectors. All experiments were repeated three times. All data are shown as mean ± SD. *P < 0.05 versus Scramble + pCMV6. ^#^*P* < 0.05 versus miR-433-3p + pCMV6.

To elucidate whether miR-433-3p influences tumor growth through regulating the expression of CREB, we constructed an overexpression plasmid of CREB (pCMV6/CREB) to perform a rescue experiment. Western blotting analysis showed that overexpression of CREB restored the protein level decreased by miR-433-3p mimics (Figure 4A). Furthermore, MTT, colony formation assays, apoptosis, cell cycle analysis, invasion and migration assays were employed in U251 and U87 cells co-transfected with miR-433-3p mimics and pCMV6/CREB or their control oligomers and vectors. As shown in Figure 4B-4G, the inhibitory effects on cell growth and invasion and migration caused by miR-433-3p were partly abolished by the overexpression of CREB (*P* < 0.05). These results demonstrate that CREB is a direct target gene of miR-433-3p and that miR-433-3p acts as a tumor suppressor through CREB in human glioma cells.

### MiR-433-3p inhibits glioma growth in nude mice

**Figure 5:**
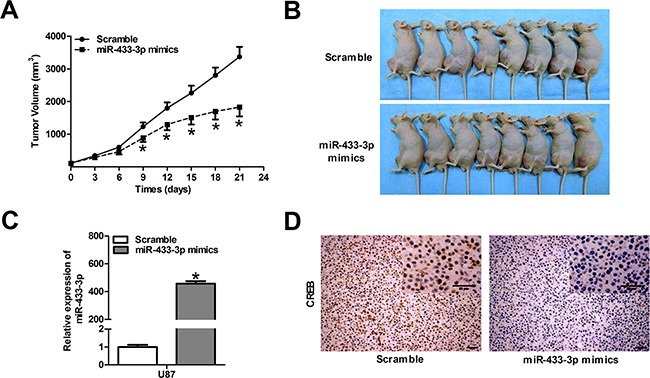
MiR-433-3p inhibits glioma growth in nude mice **A**. The U87 xenograft experiments were performed to confirm the effects of miR-433-3p on tumor growth *in vivo*. The tumor volume was measured with a caliper every 3 days using the formula, volume = length × width^2^/2. **B**. Photography of the U87 xenograft tumor was shown at the end of a 21-day observation period. **C**. The expression levels of miR-433-3p in tumor treated with miR-433-3p mimics were analyzed by quantitative RT-PCR. **D**. Immunohistochemical analysis was used to detect the expression of CREB protein in xenograft tumor after treatment with miR-433-3p mimics. Scale bars, 50μm. All data are shown as mean ± SD.*P < 0.05 versus Scramble.

Since miR-433-3p plays an important role in cell survival, we performed a proof-of-principle experiment using a U87 xenograft tumor model. Mice were treated with local injections of 500 pmol scrambled oligo or miR-433-3p mimics in 25 μl Lipofectamine at multiple sites in the tumor and were monitored every 3 days for 21 days. As shown in Figure 5A, the mean tumor volume of the mice in the treated groups was dramatically reduced at 9, 12, 15, 18 and 21 days compared to the scrambled group (*P* < 0.05). At the end of the experiment, a significant decrease in tumor volume was observed in the miR-433-3p treated group (Figure 5B), and the expression level of miR-433-3p in tumor treated with miR-433-3p mimics was significantly elevated (*P* < 0.05, Figure 5C). Besides, immunohistochemical staining revealed a down-regulated level of CREB in the miR-433-3p group (Figure 5D). The result is consistent with the effects of miR-433-3p on cultured human glioma cells *in vitro*, indicating that miR-433-3p inhibits cell growth by targeting CREB *in vitro* and *in vivo*.

### MiR-433-3p increases chemosensitivity of glioma to temozolomide (TMZ) by targeting CREB

**Figure 6:**
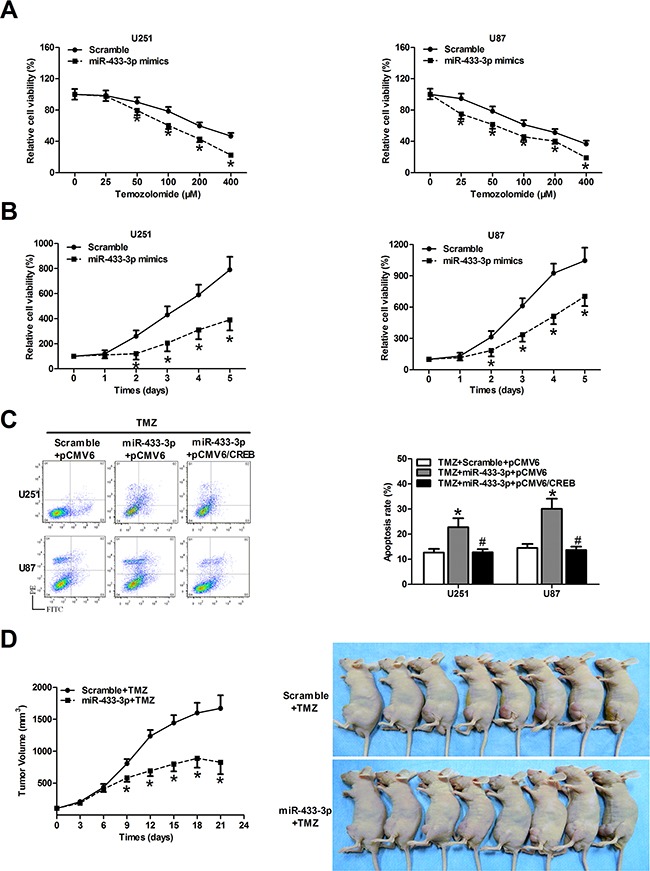
MiR-433-3p increases chemosensitivity of glioma cells to temozolomide (TMZ) by targeting CREB **A**. The cells transfected with scramble or miR-433-3p mimics were treated with different concentrations of TMZ solution, and cell survival was assayed by MTT 48 h later. *P < 0.05 versus Scramble. **B**. The cell proliferation in the presence of TMZ (100 μM) was tested by MTT every 24 h in both U251 and U87 cells overexpressing scramble and miR-433-3p. *P < 0.05 versus Scramble. **C**. U251 and U87 cells were co-transfected with miR-433-3p mimics and pCMV6/CREB or their control oligomers and vectors in the presence of TMZ (100 μM). Then flow cytometry analysis was performed to detect cell apoptosis rates in every group. *P < 0.05 versus TMZ + Scramble + pCMV6. ^#^*P* < 0.05 versus TMZ + miR-433-3p + pCMV6. **D**. Growth curve of U87 xenograft tumors and mice treated with miR-433-3p and TMZ at the end of the study. *P < 0.05 versus Scramble+TMZ. **A–C**. experiments were repeated three times. All data are shown as mean ± SD.

To investigate the potential role of miR-433-3p in chemosensitivity of glioma, U251 and U87 cells transfected with scramble or miR-433-3p mimics were treated with different concentrations of TMZ. MTT assay indicated that overexpression of miR-433-3p significantly inhibited cell viability compared with the scramble group by TMZ treatment (*P* < 0.05, Figure 6A). Furthermore, cell proliferation in the presence of TMZ was also tested by MTT at different time points. As shown in Figure 6B, we obtained consistent result that miR-433-3p increases chemosensitivity of U251 and U87 cells to TMZ (*P* < 0.05). To further explore the function of miR-433-3p and its target CREB in cell apoptosis in the presence of TMZ, U251 and U87 cells were transfected with pCMV6/CREB or pCMV6 vector. Our results showed that apoptosis rates were upregulated in the group treated with miR-433-3p and TMZ, whereas overexpression of CREB partially attenuated the effect induced by miR-433-3p and TMZ treatment (*P* < 0.05, Figure 6C). These results indicate that miR-433-3p increases chemosensitivity of glioma cells to TMZ by targeting CREB. Furthermore, the combination of miR-433-3p with TMZ significantly suppressed U87 human glioblastoma xenograft growth (*P* < 0.05, Figure 6D). These data indicate that miR-433-3p circumvents TMZ resistance of glioma both *in vitro* and *in vivo*.

## DISCUSSION

MicroRNAs (miRNAs) play a pivotal role in the development of the malignant phenotype of glioma cells, including cell survival, proliferation, differentiation, tumor angiogenesis, and stem cell generation [6]. Several recent studies conducted in human specimens point to a physiological function of miR-433 in human diseases. For instance, the level of miR-433 is decreased in human gastric carcinoma, which is associated with unfavorable outcome in overall survival, and GRB2 and KRAS are direct target of miR-433 in human gastric carcinoma [7, 14, 15]. Although these studies provide evidence for the importance of miR-433 in various human diseases, its specific function in the glioma remains elusive. In the present study, we attempt to investigate the expression and function of miR-433 in human glioma.

Previous studies have reported that miR-433 was downregulated in glioblastoma samples [16, 17]. Similarly, our data demonstrated that miR-433-3p and miR-433-5p were downregulated in human glioma tissues and 5 glioma cell lines compared with nontumor brain tissues. Therefore, we further clarified the functions of miR-433-3p and miR-433-5p in the development and progression of glioma cells. MiR-433-3p mimics and miR-433-5p mimics were used to transfect human glioma cell lines U251 and U87. The up-regulation of miR-433-3p dramatically inhibited glioma cell proliferation, repressed invasion and migration and promoted cell apoptosis, which is consistent with its potential roles as a tumor suppressor in glioma. We also used xenograft models to confirm that miR-433-3p could suppress the growth of glioma cell *in vivo*. These data suggest that miR-433-3p rather than miR-433-5p might play a critical role in the development of glioma and act as a tumor suppressor.

The role of miRNA in the organism is determined by the target genes. Therefore, it is important to identify the target genes of miR-433-3p to study its mechanism. In our study, CREB was identified as a direct target of miR-433-3p in U251 and U87 cells. Besides, overexpression of CREB rescued the phenotypes caused by miR-433-3p, indicating that CREB is regulated by miR-433-3p in glioma cells. CREB (cAMP response element-binding protein, or CREB1) is a nuclear transcription factor which contains a basic/leucine zipper structure (bZIP) [18, 19]. It can be activated through phosphorylation at Ser-133 via a number of kinases pathways, including the PKA, PKC, CaM kinases, p90RSK, and ERK1/2 signaling pathways [20–22]. It has been reported that CREB is a real culprit in oncogenesis [23]. CREB has been implicated in the growth and progression of multiple cancers, including malignant mesothelioma [22], non-small cell lung cancer [24, 25], leukemia [26, 27] and breast cancer [28]. CREB is also highly-expressed in glioma tissues and promotes the growth and survival of glioma cells [29–32]. The mechanism by which CREB regulates glioblastoma tumor cell proliferation involves activities downstream from both the MAPK and PI3K pathways that then modulate the expression of three key cell cycle factors, cyclin B1, cyclin D1 and PCNA [32]. It has been shown that miR-1224-5p and miR-200b act as tumor suppressors by targeting CREB in malignant gliomas [33, 34]. Herein, we demonstrated that CREB is a direct target of miR-433-3p in glioma cells. What's more, consistent with the functional regulation of CREB by miR-433-3p, the protein expression of PCNA, BCL2, CyclinD1 and MMP-9 were suppressed by miR-433-3p in the present study. These findings indicate that CREB is downregulated by miR-433-3p, and that CREB subsequently directly or indirectly modulates its target genes to control the cell growth and metastasis in glioma.

We then further explored the role and mechanisms of miR-433-3p in chemosensitivity of glioma. Emerging evidence suggests that miRNA regulates chemosensitivity of glioma through apoptosis, cell cycle distribution and cancer stem cell (CSC) maintenance [35–37]. Our results showed that apoptosis rates were upregulated in the group treated with miR-433-3p and TMZ, whereas overexpression of CREB partially attenuated the effect induced by miR-433-3p and TMZ treatment. These results implicate that miR-433-3p increases chemosensitivity of glioma to TMZ through targeting CREB to activate apoptotic signaling pathways. Some studies reported that reduced expression of CREB suppressed the expression of ABCG2 [38], which plays critical roles in conferring multidrug resistance in tumor cells [39–41]. Therefore, potential mechanistic explanations for the TMZ chemosensitivity of miR-433-3p in glioma may be due to apoptosis activation, cell cycle distribution and downregulation of CREB and ABCG2.

In summary, our data reveal that miR-433-3p is downregulated in glioma tissue and cells, and functions as a tumor suppressor by targeting CREB in glioma, thus regulating cell growth, invasion and migration. Besides, we demonstrate that miR-433-3p renders glioma cells more susceptible to TMZ treatment. Consequently, our findings suggest that miR-433-3p may function as a potential target in diagnostic and therapeutic procedure in malignant glioma.

## MATERIALS AND METHODS

### Cell culture and transfection

The human GBM cell lines U251, U87, LN229, SNB19 and LN308 were purchased from the Institute of Biochemistry and Cell Biology, Chinese Academy of Sciences, Shanghai, China. The cells were maintained in Dulbecco's modified Eagle's medium (DMEM, Gibco, USA) supplemented with 10% fetal bovine serum (FBS, Gibco, USA), and incubated at 37°C with 5% CO_2_. The oligonucleotide sequence of human miR-433-5p mimics (5′-UACGGUGAGCCUGUCAUUAUUC-3′) and human miR-433-3p mimics (5′-AUCAUGAU GGGCUCCUCGGUGU-3′) were synthesized by Gima Biol Engineering Inc., Shanghai, China. A scrambled oligomer sequence (5′-UUCUCCGAACGUGUCAC GUTT-3′) was used as the negative control. Transfection was performed with Lipofectamine 2000 Reagent (Invitrogen, USA) following the manufacturer's protocol.

### Clinical tumor speciments

Twelve glioblastoma tissue samples were collected in Tianjin Huanhu Hospital from January 2013 to May 2013. Six nonneoplastic brain tissues were obtained from patients with traumatic brain injury as control. Immediately after surgery, samples were snap-frozen and stored in liquid nitrogen. The patient clinical information is shown in Table 1. This study was approved by the ethics committee of Tianjin Huanhu Hospital and informed consent was obtained according to the Declaration of Helsinki.

**Table 1:** Clinical information of patients and control subjects

Patients				Control subjects			
Index	Gender	Age	WHO grade	Index	Gender	Age	WHO grade
1	Male	34	IV	1	Male	23	Normal brain tissue
2	Female	46	IV	2	Female	44	Normal brain tissue
3	Female	38	IV	3	Male	36	Normal brain tissue
4	Female	36	IV	4	Female	33	Normal brain tissue
5	Male	38	IV	5	Male	28	Normal brain tissue
6	Male	39	IV	6	Male	46	Normal brain tissue
7	Male	46	IV				
8	Female	48	IV				
9	Male	30	IV				
10	Male	42	IV				
11	Male	41	IV				
12	Male	41	IV				

### Real-time PCR for miR-433-5p and miR-433-3p expression

Total RNA from tissue samples and glioma cells were extracted using a miRcute miRNA Isolation Kit (Tiangen Bio Co., Ltd, Peking, China) following the manufacturer's instructions. Synthesis of complementary DNA (cDNA) and real-time polymerase chain reaction (RT-PCR) were performed using the PrimeScript^®^ RT reagent Kit and SYBR^®^ Premix Ex Taq^TM^ II (Takara Co., Ltd, Dalian, China), respectively according to the manufacturer's protocol. Real-time PCR was performed using Roche LC480 quantative Real-Time PCR system (Roche, USA). All PCR reactions were performed using standard PCR conditions according to reagent Kit. U6 was used as the internal control. The expression of each gene was quantified by measuring cycle threshold (Ct) values and normalized using the 2^−ΔΔCt^ method relative to U6 snRNA. The primer sequences were synthesized by Invitrogen Co., Ltd, Shanghai, China.

### MTT and colony formation assays

The 3-(4, 5-dimethylthiazole-2-yl)-2, 5-biphenyl tetrazolium bromide (MTT) assay was used to evaluate cell viability and proliferation. U251 and U87 were seeded in a 96-well plate at 5,000 cells per well 12 hour prior to transfection. The cells were transfected with miRNA mimics or a scrambled siRNA at 3pmol per well. The MTT assay was used to determine cell viability 24 h, 48 h and 72 h after transfection. The spectrophotometric absorbance of each sample was measured at 490 nm.

For the colony formation assay, tumor cells transfected with miRNA mimics or a scrambled oligomer were counted and seeded in 12-well plate at 100 cells per well. The cells were grown for 10 days at 37°C with 5% CO_2_. Culture medium was replaced every 3 days. Colonies were counted only if they contained more than 50 cells, and the number of colonies was determined at the sixth day after seeding. Colony formation was visualized with crystal violet staining.

### Apoptosis assay and cell cycle analysis

U251 and U87 cells were plated into 6-well plates and transfected with oligonucleotides. Apoptosis was measured 48 h after transfection by using an Annexin V/fluorescein isothiocyanate (FITC) apoptosis detection kit (BD Biosciences, USA). Briefly, cells were trypsinized, washed, stained with FITC-conjugated anti-Annexin V antibody under darkness for 15 min at room temperature, and then analyzed by FACSCanto II flow cytometry (BD Biosciences, USA). Each experiment was performed in triplicate.

The cell cycle was evaluated 48 h after mimics transfection by FACSCanto II flow cytometry (BD Biosciences, USA). Briefly, a total of 1 × 10^6^ cells were harvested, rinsed with cold PBS and fixed with 70% ice-cold ethanol for 48 h at 4°C. Fixed cells were rinsed with cold PBS, and their nuclei were stained with propidium iodide (PI) before DNA content was measured with flow cytometry. At least 10,000 cells were counted, and the ModFit software was used to analyze cell cycle distribution.

### Migration and invasion assays

For the transwell migration assays, 5 × 10^5^ U251 and U87 cells were respectively plated in the top chamber with a noncoated membrane (Costar, USA). For the invasion assays, 1 × 10^5^ U251 and U87 cells were respectively plated in the top chamber with a Matrigel-coated membrane. For both assays, the cells were plated in the upper chambers filled with serum-free medium, and medium supplemented with 20% serum (Gibco, USA) was used as a chemo-attractant in the lower chamber. The cells were incubated at 37°C with 5% CO_2_ in a tissue culture incubator. After 16 h, the non-migrated/non-invaded cells were removed from the upper sides of the transwell membrane filter inserts using cotton-tipped swabs. The migrated/invaded cells on the lower sides of the inserts were stained with crystal violet, and five random fields of the cells were counted.

### Prediction of miR-433-3p target gene and Luciferase reporter assay

Using TargetScan, PicTar, and miRanda, we predicted multiple putative targets of miR-433-3p based on the conserved seed region between miR-433-3p and the 3′-UTR of each gene. Based on the bioinfomatic analysis by three computational algorithms, we found that CREB, PPM1A, and KRAS were the predicted targets of miR-433-3p. The 3′-UTR target sites of the predicted target gene and a mutant variant were synthesized and cloned in the pmirGLO Dual-Luciferase miRNA target expression vector. The new vectors were named pmirGLO-CREB-3′UTR-Wild/Mut, pmirGLO-PPM1A-3′UTR-Wild/Mut and pmirGLO-KRAS-3′UTR-Wild/Mut.

To examine the luciferase activity, 4 groups were set up for U251 and U87 cells, respectively, (1) Scramble + Mut, (2) Scramble + Wild, (3) mimics + Mut, (4) mimics + Wild. Glioma cells were seeded in 96-well plates and co-transfected 12 h later with plasmids (0.2 μg), miR-433-3p mimics (5 pmol) or scramble miRNA (5 pmol) using Lipofectamine 2000 (Invitrogen, USA). The cells were harvested 48 h after transfection, and firefly and renilla luciferase activities were analyzed with the Dual-Luciferase^TM^ Reporter Assay System, according to the manufacturer's instructions (Promega, USA).

### Western blot analysis

Total cellular proteins were extracted and separated by SDS-PAGE gels, and Western blot analysis was performed according to standard procedures. β-actin on the same membrane was used as a loading control. The primary antibodies included anti-CREB (CST, USA), and anti-PCNA, anti-BCL2, anti-CyclinD1, anti-MMP-9 (SantaCruz, USA). Proteins were visualized using the ECL procedure (Millipore, USA). The bands of gray intensity were analyzed by Image J software.

### Xenograft experiments

Male athymic BABL/c nude mice (4 weeks old) were purchased from the Animal Center of Chinese Academy of Science (Shanghai, China). All the animal experiments were carried out in the animal unit, Tianjin Medical Universtity (Tianjin, China) according to procedures authorized and specifically approved by the institutional ethical committee (Permit Number: SYXK 2009-0001). The U87 subcutaneous tumor xenograft model was established before experiment. When the tumors were approximately 100 mm^3^ in volume, the mice were randomly divided into 2 groups (8 mice per group): the scramble-treated group and the miR-433-3p mimics-treated group. These mice were then treated with 500 pmol miR-433-3p mimics (20 pmol/μl) or scramble miRNA (20 pmol/μl) in 25 μl Lipofectamine through a local injection at 4-5 sites of the xenograft tumor. When the mixture of Lipofectamine 2000/mimics was injected into the xenograft tumor in a multisite injection manner, the needle should be kept under the skin to prevent the leakage of the liquid. Treatment was conducted every 3 days, until the end of the experiment. The tumor volume was measured with a caliper every 3 days using the formula, volume = length × width^2^/2. At the end of a 21-day observation period, the mice were sacrificed to detect the change of miR-433-3p and CREB expression *in vivo*. The tumor tissues removed from the mice were stored in liquid nitrogen and formalin for RT-PCR and immunohistochemical analysis according to standard procedures.

The same method was used to detect the potential role of miR-433-3p in chemosensitivity of glioma in U87 xenograft tumor model. The mice were also randomly divided into 2 groups (8 mice per group): the scramble + TMZ group and the miR-433-3p mimics + TMZ group. Temozolomide (7.5mg/kg/day) was administered by intraperitoneal injection every 3 days. The mixture of Lipofectamine 2000/mimics was injected into the xenograft tumor in a multisite injection manner. Tumor volume was calculated at length × width^2^/2.

### *In vitro* chemosensitivity assay to temozolomide (TMZ)

U251 and U87 cells were plated into 96-well plates at a density of 5000 cells per well 12 hour prior to treatment. The cells transfected with scramble or miR-433-3p mimics were treated with different concentrations of TMZ solution, and cell survival was assayed by MTT 48 h later. Besides, the cell proliferation in the presence of TMZ (100 μM) was also tested by MTT every 24 h in both U251 and U87 cells overexpressing scramble and miR-433-3p. To further explore that the mechanism of miR-433-3p increasing chemosensitivity to TMZ in glioma, U251 and U87 cells were co-transfected with miR-433-3p mimics and pCMV6/CREB or their control oligomers and vectors in the presence of TMZ (100 μM). Then flow cytometry analysis was performed to detect cell apoptosis rates in every group.

### Statistical analysis

SPSS 16.0 software was used for the statistical analysis. One-way ANOVA or Student's t-test was used for comparisons between groups. Differences were considered significant at *P* < 0.05.
